# Behavioural Differences between Single Scandinavian Brown Bears (*Ursus arctos*) and Females with Dependent Young When Experimentally Approached by Humans

**DOI:** 10.1371/journal.pone.0121576

**Published:** 2015-04-01

**Authors:** Veronica Sahlén, Andrés Ordiz, Jon E. Swenson, Ole Gunnar Støen

**Affiliations:** 1 Department of Ecology and Natural Resource Management, Norwegian University of Life Sciences, Ås, Norway; 2 Grimsö Wildlife Research Station, Department of Ecology, Swedish University of Agricultural Sciences, Riddarhyttan, Sweden; 3 Norwegian Institute for Nature Research, Trondheim, Norway; 4 Department of Wildlife, Fish and Environmental Studies, Swedish University of Agricultural Sciences, Umeå, Sweden; University of Hawaii at Manoa, UNITED STATES

## Abstract

Carnivore-human encounters that result in human injury present a conservation and management challenge and it is therefore important to understand under what conditions such incidents occur. Females with cubs are often involved when humans are injured by brown bears *Ursus arctos*. In Scandinavia, this is particularly true for unarmed recreational forest users. Our aim was to document behavioural differences between single bears and females with cubs in order to develop recommendations to minimize the risk of injuries to recreational forest users. We documented the reactions of GPS-collared females with cubs and single brown bears to experimental approaches by humans to 50 m from the bear on 42 and 108 occasions, respectively. The majority of females with cubs (95%) and single bears (89%) left when approached. Bears that left were passed at shorter distances and were in more open areas than those that stayed. Both groups had similar flight initiation distances, which were longer for bears that were active at the time of the disturbance. Females with cubs selected more open habitat than single bears, also for the new site they selected following disturbance. Females with cubs, particularly active females with cubs of the year, moved greater distances and spent more time active following the approach. Females with cubs and single bears were seen or heard in 26% and 14% of the approaches, respectively. None of the bears displayed any aggressive behaviour during the approaches. Females with cubs selected more open habitat, perhaps predisposing them to encountering people that are not involved in hunting activities, which might be the primary explanation why females with cubs are most frequently involved when unarmed people are injured by bears in Scandinavia. To mitigate injury risks, one must consider factors that bring bears closer to human activity in the first place.

## Introduction

Large carnivores present a particular conservation and management challenge, as they often cause damage to livestock and/or property, as well as cause fear due to the potential risk they pose to humans [1,2]. Past management strategies to eliminate these threats resulted in major population declines among large carnivores in both Europe and North America [3]. After a change to more conservation-oriented management, many large carnivore populations have increased [4,5,6] and combined with expanding human populations and increasing backcountry use, the chance of large carnivore-human encounters have also increased [7,8], thus affecting humans [9].

The brown bear *Ursus arctos* population in Scandinavia has increased dramatically in recent decades [10], with a concomitant increase in human injuries caused by brown bears, including two fatalities during the 2000s [11], and public acceptance for the bear is decreasing [12]. The increase in bear-caused human injuries has occurred among armed men mainly involved in hunting activities, whereas no such increase has been detected for unarmed people [11]. For unarmed people, however, the greatest risk factor is encountering females with cubs [11]. Of the 8 incidents involving unarmed people since 1977, females with cubs have been involved in 6 incidents [11,13].

Brown bears generally avoid people and human activities throughout their range, both spatially and temporally [7,14]. Experimental approaches on single brown bears in Scandinavia have also shown that they avoid confrontation with people [15]. The bears’ responses to the disturbance were immediate and pronounced and the effects on their movement patterns lasted several days [16]. The effects of disturbance on animals could be similar to that of predation risk. Indeed, bears adjust their behaviour to avoid human encounters in a way similar to prey avoiding their predators [7,17], which has also been documented in lions *Pantera leo* and wolves *Canis lupus* [18,19].

In this study, we experimentally approached females with cubs to document their responses to human disturbance. The results are of interest to the general public and management agencies, both because human disturbance has potential fitness consequences that could be particularly problematic for females with cubs [16], whose survival rates are important for population trends [20], and because understanding the responses of females with cubs to humans is key to prevent incidents involving unarmed people. The overall aim was therefore to document the behaviour of females with cubs during encounters with humans and compare with single bears in order to explain why females with cubs are more often involved when unarmed people are injured by bears in Scandinavia [11].

The risk-disturbance hypothesis in the context of parental investment predicts that the response to disturbance should be greater in individuals with dependent offspring, which could cause abandonment of the young if they are unable to follow their mother [21]. However, bear cubs, and particularly yearlings, are quite mobile during summer and autumn. The presence of cubs may therefore not keep a female from leaving due to human disturbance, even if the move may incur energetic costs for the cubs. We therefore hypothesized that females with cubs would respond to approaching humans by leaving more often and at longer distances than single bears, and that they would respond to disturbance by moving farther and remaining active for longer.

## Materials and Methods

### Study area

The study area is located in south-central Sweden (Dalarna and Gävleborg counties), (61°N, 14°E). The topography is varied, with gently rolling hills, and >90% of the area is below the timberline (~750 m a.s.l.) [22]. The forestry industry manages the area intensively, resulting in a large proportion of young forest and interspersed clear-cuts (Swenson et al. 1999a). Scots pine (*Pinus sylvestris*) and Norway spruce (*Picea abies*) dominate the landscape, with some deciduous trees, consisting mainly of birch (*Betula pendula* and *B*. *pubescens*), mountain ash (*Sorbus aucuparia*) and willows (*Salix* spp.) [23]. Forestry practices have also created an extensive road network of gravel roads of varying sizes and paved public roads [14]. The area is popular for berry pickers and other recreational users, including hunters.

### The bears and approaches

#### Approach method

All bears we studied, except dependent young, were equipped with a GPS Plus-3 or GPS Pro-4 collar (VECTRONIC Aerospace GmbH, Berlin, Germany) and a VHF transmitter implant (IMP400L) (Telonics, USA). All yearlings were equipped with a VHF transmitter implant. The bears were captured and handled during March—May using the methods for marking and capturing described earlier [24,25]. The captures of the bears were approved by the Swedish Environmental Protection Agency (permit Dnr 412-7327-09 Nv) and the approaches by the Ethical Committee on Animal Experiments in Uppsala, Sweden (permit C 47/9).

The GPS-collars were programmed via the GSM network to fix a location every minute for a 3-hour period during which the approach was conducted; 1 hour before the approach started (control period), 1 hour during, and 1 hour after the approach. The data were delivered via the GSM network to our database and any omitted positions were later physically downloaded from the collar, once it had been retrieved. We used the approach method first described in [15,16]. Prior to the approach start, observers located females with cubs using a combination of triangulation of the VHF signal and last received GPS positions sent directly to the observers’ cell phones. We standardized the experiment and aimed to pass the bears at a distance of 50 m with the wind direction toward the bear at the time of passing, so it would be able to catch wind of us as we passed. Observers talked with each other in a normal speaking voice during the approach or to him/herself if he/she was alone. Approaches were conducted between 9:00–14:00 hrs local time (GMT+2) by 2 observers in most cases (median = 2, range: 1–6, N = 150). Specifically, 29 approaches were conducted by 1 observer, 96 by 2, 21 by 3, and 2 each by 4 and 6 observers. The observers’ track was recorded using a hand-held GPS receiver (Garmin GPSMAP 60CSx (Garmin Ltd, USA) which was programmed to record one position every 10 m.

The majority of single bears left after being disturbed [15]; thus, for ethical reasons we did not approach females with cubs during the main breeding season in early summer, as their displacement could expose the cubs to increased risk of infanticide. Infanticide is a prominent cause of death for subadult bears in Scandinavia, particularly during the mating season, which occurs mid-May until mid-July [26], and all approaches were therefore conducted from the second half of July until mid-October.

The approach method was developed based on available information on risk factors associated with attacks, statistics on the frequency of attacks in Scandinavia, a review about bear behaviour in encounters with research personnel [27,28] and the cumulative experience of project staff and researchers about bear behaviour during encounters based on almost 30 years of field-based research in the area. Based on this information, the overall risk of an attack during an approach was considered to be extremely low. The addition of any physical safety measure (such as bringing firearms or pepper spray) was considered to, if anything, increase the risk of injury to the observers. Instead, using a normal speaking voice to alert bears of approaching humans was considered a preventative safety measure and was a part of the standardized method. In addition, all fieldwork personnel received training in bear behaviour and how to behave in bear encounters under various conditions.

#### Females with cubs

We conducted 42 approaches on females with cubs (19 on females with cubs of the year (FCOY) and 23 on females with yearlings (FY) in 2008–2011 (2008, 9; 2009, 15; 2010, 12; and 2011, 6). Two females with cubs were approached in more than one year, and the total number of individual females was 12. We waited a minimum of 21 days between consecutive approaches to females with cubs. Each female with cubs was approached on average 3 occasions per year (range 1–5) and 3.5 times in total during the study (range 1–7). Females were 6–16 years old and were accompanied by one, two, or three cubs (2.9 ± 0.6 cubs (mean ± SD)).

We had a GPS fix success rate of 93.3 ± 15.1%. The observers started the approaches 1013.0 ± 995.6 m from the family group and walked at a normal hiking pace of 3.5 ± 1.5 km·h^-1^.

#### Single bears

We approached 23 female and eight male single bears on 108 occasions (76 on females and 32 on males) in 2006–2009 (2006, 10; 2007, 40; 2008, 48; and 2009, 10). Single bears were approached throughout the whole season and on average 3.5 occasions per individual per year (range 1–6), but we used only data from the equivalent period as for females with cubs. The mean GPS fix success rate was 66% for each year, but this improved greatly between 2006 (37 ± 12%) and 2009 (98 ± 1%), mainly due to improvements in technology [15]. The approaches started at 869 ± 348 m from the bears and observers kept a normal hiking pace of 3.4 ± 0.6 km·h^-1^.

### Data analysis

We classified females with cubs as either passive (resting at daybeds) or active (moving, foraging, etc), based on their activity during the control period until the start of the approach [15]. This resulted in 12 active and 30 passive females with cubs and 23 active and 84 passive single bears. We calculated an upper control limit (UCL) for speed (m·min^-1^) for bears that were either active or passive during the entire control period using statistical quality control [29], against which we could measure potential reactions to disturbance in terms of flight initiation distance (FID). This is referred to as statistical process control and is commonly used for controlling industrial processes [30]. First, an “in control” or normal behavioural process is identified (in this case normal speed/movement pattern) and control limits are calculated using mean and standard deviation. When the process runs outside the defined control limit (in this case exceeding the UCL), the process is said to be “out of control”. We were able to determine FID for 36 family groups and 62 single bears.

Initial analyses of average speed revealed a difference in speed between females with cubs and the single bear dataset, so we tested the differences using Welch’s t-test to account for unequal variances and sample sizes, after log-transforming the speed to normalize the residuals (log(speed*100)). Active females with cubs had higher speed (12.4±12.2 m/min, n = 943) than active single bears (11.4±17.4 m/min, n = 1359) (t_α = 0.05,df = 2222.097_ = 8.2664, *P*<0.001), but there was no difference between passive females with cubs (5.0±7.7 m/min, n = 1218) and passive single bears (5.6±7.6 m/min, n = 4703) (t_α = 0.05,df = 1837.976_ = -6.3696, *p* = 0.239). We therefore calculated a new UCL for active females with cubs and pooled the passive females with cubs and passive single bear approaches to calculate a new UCL for passive bears. UCL was estimated to 41.5 m/min for passive bears, against which passive females with cubs were assessed and 127.4 m/min for active females with cubs, against which active females with cubs were assessed. The single bears were assessed against the UCL previously established for active single bears (see [15]).

The position immediately preceding the first point at which UCL was exceeded was considered the bear’s flight initiation point, and the distance between this point and the temporally corresponding observer location was considered the FID. We did not estimate FID if there had been more than 1 minute between the point at which UCL had been exceeded and the immediately preceding point. We checked the accuracy of the method by visual inspection of the dataset using ArcGIS 10.x (ESRI, 2010).

There were two factors that made an approach unsuitable for FID analyses; 1) the bear did not exceed UCL during the approach, or 2) exceeding UCL was delayed (i.e. FID occurred later than the movement suggested). Where bears did not exceed UCL, we could not determine whether they left because of our disturbance, so we removed them from all further analyses pertaining to reaction to disturbance. In some instances, passive bears exceeded UCL some minutes after leaving the initial site. As FID was identified based on a standardized UCL, this created a time lag between leaving the site (visible reaction) and the flight initiation as determined by exceeding the UCL. This resulted in a higher degree of uncertainty in the FID for these approaches compared to the other approaches where this time lag was not observed. We therefore excluded the approaches with a time lag from analyses involving FID, but as we considered the bears to have responded to our disturbance, they were included in analyses that were not dependent on FID.

We examined whether bears stayed or left, the FID for those that left, and we visited the sites where the bear had been prior to the approach (initial site) and the new site where the bears settled after being approached (second site) to estimate the horizontal vegetation cover. We used sighting distance as a way to measure horizontal vegetation cover; the more vegetation cover, the lower the sighting distance. We placed a two-coloured cylinder at the initial and second sites, measured the maximum distance in each cardinal direction from which the cylinder could still be seen and averaged this distance to obtain the sighting distance [15,31]. We also recorded the habitat type at initial and second sites using forestry habitat classification [32,33] (Table 1). We tested if observations (seeing and/or hearing) of single bears and females with cubs differed in frequency using a Chi-square test of association with Yates’ Correction for Continuity at α = 0.05.

**Table 1:** Forestry habitat classifications used in the description of initial and second sites of Scandinavian brown bears approached by humans on foot in Sweden (in alphabetical order by code).

***Habitat type***	***Description***
G—Mid-aged forest	Refers to G1/G2 in Karlsson & Westman (1991). Medium tree ³ 10 cm diameter at breast height
R—Pre-commercial thinning	Refers to R1 and R2 in Karlsson & Westman (1991).Young forest; primary, non-commercial thinning. Tree sizes range from planting stage until medium tree is >1.3 m but < 10 cm in diameter at breast height
RD—Road	Road (irrespective of size, type or condition)
S—Mature forest	Refers to S1 in Karlsson & Westman (1991). Mature forest at the age when ca 10 years remain before the final harvest, and older (in our area an S1 forest is 80–90 years and above)
SF—Swamp forest	Swamp forest—a waterlogged ground (not on peat), often with broadleaf grasses and herbs and sedges, with trees. Contrary to the bog, in a “swamp” there is in- and outflow of groundwater that adds to the productivity, and a “swamp” often has some herbs that demand high productivity.
TRB—Tree rich bog	Like a bog (very wet ground, on peat ground with low productivity and no in-or out flow of ground water, with no or very few trees) but more rich in trees.

We assessed the factors affecting sighting distance, whether bears stayed or left, their FID, time spent active and distance moved after the approach using generalized linear mixed models (GLMMs) in the lmer/glmer package (lme4 library, R Development Core Team 2011), with bear ID as a random variable to account for the repeated sampling of individuals. To identify the best candidate models, we used model dredging [34,35] with the model.dredge package (MuMIn library, R Development Core Team 2011). In contrast to the stepwise regression approach, which works by examining one variable in turn and removing uninfluential variables and interactions until one final model with only significant variables remains, model dredging works by running all possible combinations of the input explanatory variables and calculating Akaike’s Information Criterion (AIC) values for each combination (candidate model) [36]. In our case, corrected AIC (AIC_C_) values were calculated to account for small sample sizes (Sugiura 1978: in [36]). All possible models can then be ranked according to their AIC/AIC_C_ value, where the lowest value signifies the best model [37]. Model dredging has been criticized as a “fishing expedition”, which can produce spurious results [37]; however, such an approach can be useful for observational studies, e.g. [34,35]. When using an information-theoretic approach, it is important to select explanatory variables with care, as the results must always be considered in relative terms (i.e. selection of variables with little biological relevance could still generate a “best model”). Because all variables in our full models had a biological rationale and support from prior research in this study area, we are confident of their biological/ecological relevance, which further reduced the risk of finding nonsensical candidate models. Model selection using AIC/AIC_C_ typically identifies the model with the lowest value as the best model [37]. However, models that do not differ from the best model by more than 2 AIC units (i.e. ΔAIC <2) are likely to be equally good [37]. Therefore, rather than selecting one best model based on the lowest AIC_C_ value, we selected the best candidate models with ΔAIC_C_ < 2 from the model dredging. We then calculated AIC_C_ weights for the best candidate models, as well as for the variables included in the models [38], which is a form of model averaging. Candidate model weights provide the probability that a model is the best model, and dividing the weights of two candidate models will give the relative strength of one model over another [38]. Variable weights provide information on how often an explanatory variable has been included in the selected candidate models (i.e. a variable that is included in all candidate models will have a variable weight of 1), which gives the relative importance of each variable [35].

For the analyses, we constructed full models for each of the response variables under investigation, including potential explanatory variables and interactions from previous findings [15], preliminary exploratory analyses, and what we considered biologically reasonable. We included only interactions in the models if initial data exploration revealed it was warranted. Sex was not a significant factor in Moen et al. [15] or in Ordiz et al. [16], so all single bears were grouped together. Initial data exploration did not show any great differences between females with cubs of the year (FCOY) and females with yearlings (FY), and these were grouped together as females with cubs in the analyses (variable Reproductive category; females with cubs or single). There was one exception; we analysed them as separate categories (here Family status: FCOY, FY or single) for the behaviour after the approach (distance moved and time spent active following the approach). Based on the above-mentioned considerations, other factors that could potentially influence bear behaviour (staying/leaving, FID and behaviour after disturbance) when approached by humans were the bear’s age, if the bear was active or resting in a daybed, the vegetation cover around the bear’s location, how many observers conducted the approach, and their passing distance. It was also reasonable to expect that a higher number of observers at a close passing distance could have a larger effect than a farther distance or fewer observers, and that this in turn also could depend on how well the bear was hidden at the time (vegetation cover). Which variables and interactions were included depended on the behaviour under investigation. In all models, variables were transformed for normalisation where applicable. The full models for the respective response variables were therefore as follows:

Sighting distance ~ bear reproductive category * bear activity * site type (initial or second site) * habitat type

Bears stayed or left after the approach ~ bear age * bear activity * bear reproductive category + sighting distance * observer passing distance * number of observers

FID ~ bear age * bear activity * bear reproductive category + sighting distance* number of observers

Distance moved after the approach ~ bear activity * family status + sighting distance * observer passing distance + number of observers * observer passing distance

Time spent active after the approach ~ bear activity * family status + sighting distance * observer passing distance * number of observers

The “~” symbol indicates the relationship between the response variable and the explanatory variables included in the model. The “+” symbol indicates inclusion of a variable in the models without an interaction with other variables, whereas the “*” symbol indicates interactions between the variables included.

## Results

We passed the bears’ initial sites at 50.6 ± 53.7 m (n = 146, mean ± SD). We heard or saw females with cubs in 11 of 42 (26%) approaches (6 FCOY, 5 FY) and single bears in 15 of 108 approaches (14%) (10 females, 5 males). This difference was not statistically significant, but suggests a trend for females with cubs to be more easily detected than single bears (χ^2^_(1, N = 150)_ = 3.00, *P* < 0.10). None of the bears showed any aggression toward the observers.

**Fig 1:**
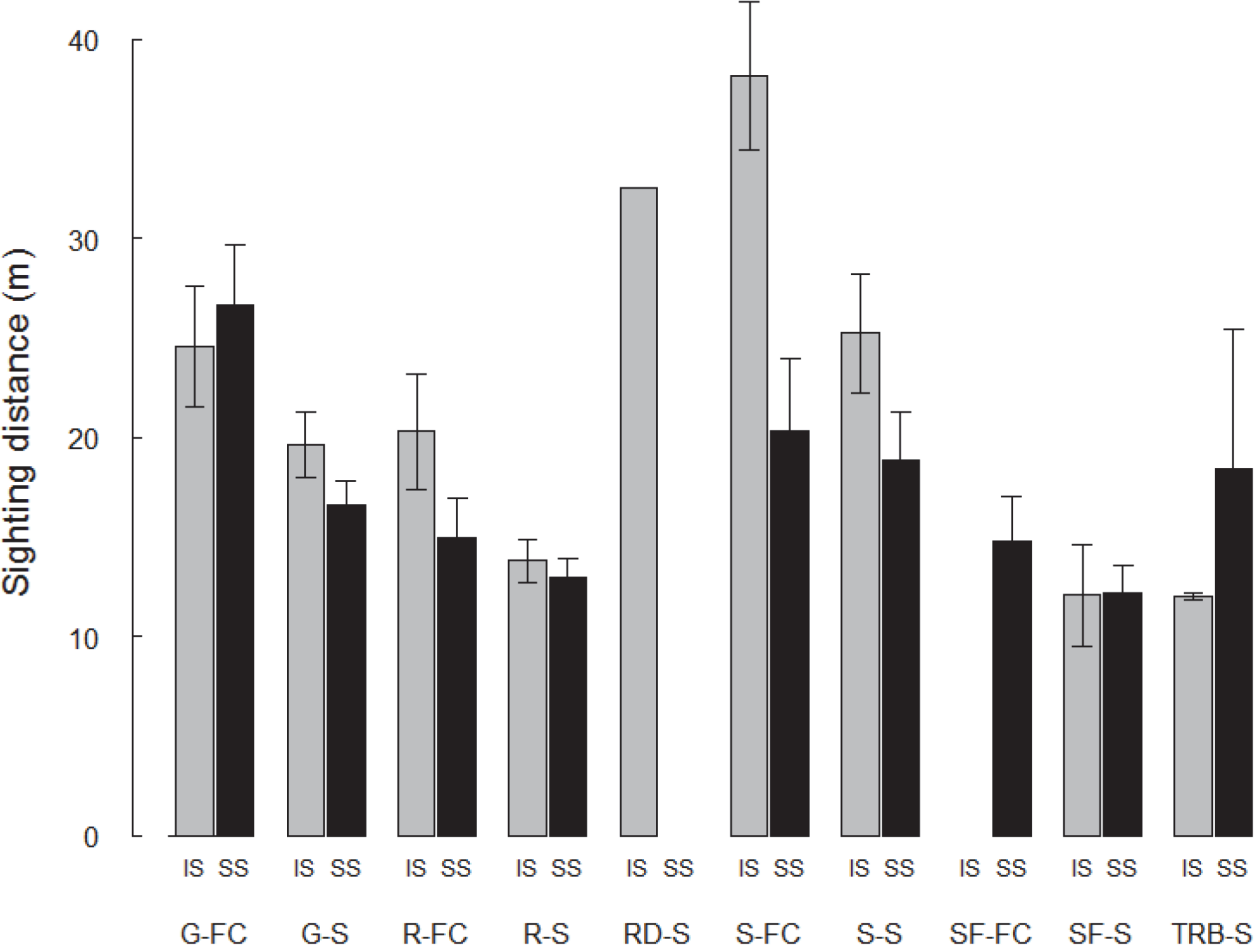
Sighting distance at initial and second sites for brown bears in Scandinavia after being approached. Mean sighting distance (m) at initial (IS) and second sites (SS) for approached brown bears in different habitat types in Scandinavia (see Table 1 for habitat type definitions), presented for females with cubs (FC) and single bears (S), in order of habitat type (e.g. G-FC = mid-aged forest, females with cubs). Longer sighting distance indicates less horizontal vegetation cover (i.e. more open habitat). The error bars show the standard error of the mean.

Both initial and second sites of females with cubs had longer sighting distance (less cover) than those of single bears, regardless of habitat type (Fig 1). Sighting distance was longer for active bears than passive bears, longer in initial than second sites, and differed among habitat types (Table 2).

**Table 2:** Best models explaining sighting distance for brown bears that were approached by humans in Sweden.

Model							AIC_C_	ΔAIC_C_	W_*i(M)*_
1	Activity + habitat + site type + reproductive category						316.5	0	0.7
2	Activity + habitat + site type + reproductive category + activity*reproductive category						318.2	1.72	0.3
W_i(V)_:	Activity (1.0), habitat (1.0), site type (1.0), reproductive category (1.0), activity * reproductive category (0.3)								

Resulting candidate models with ΔAIC_C_ < 2 after model dredging. We show AIC_C_ values, differences in AIC_C_ values (ΔAIC_C_) and AIC_C_ weights for models (W_*i(M*)_) and variables (W_*i(V)*_). The “+” symbol indicates inclusion of a variable in the models without an interaction with other variables, whereas the “*” symbol indicates interactions between the variables included.

Whether the bears stayed or left was affected by passing distance (i.e. the closest distance between the observers’ track and the initial site) and sighting distance at the initial site. It was also affected by the number of observers, where all bears left during approaches with 3 or more observers, compared to 86 and 87% for 1 and 2 observers, respectively. Staying or leaving was affected to a lesser extent (as indicated by lower variable weights), by reproductive category, age and activity (Table 3). The passing distance was shorter for bears that decided to leave, which were also in more open areas (longer sighting distance) than those that stayed. There was a tendency for females with cubs to be more likely to leave than single bears and for younger bears to be more likely to leave than older bears. The FID was primarily affected by the bears’ activity with longer FID for active bears than passive bears. There was a weaker effect of age, where younger bears had longer FID than older bears, as well as a smaller effect of the number of disturbers (Table 4).

**Table 3:** Best models explaining whether brown bears stay or leave after being approached by humans in Sweden.

Model										AIC_C_	ΔAIC_C_	W_*i(M)*_
1	Passing distance + sighting distance + number of observers									86.80	0.00	0.14
2	Passing distance + sighting distance									87.10	0.24	0.13
3	Age + passing distance + sighting distance + number of observers									87.50	0.66	0.10
4	Passing distance + sighting distance + reproductive category									87.90	1.04	0.09
5	Age + passing distance + sighting distance									87.90	1.10	0.08
6	Age + passing distance + sighting distance + reproductive category									88.10	1.30	0.08
7	Activity + passing distance + sighting distance + number of observers									88.30	1.46	0.07
8	Passing distance + sighting distance + number of observers + reproductive category									88.30	1.48	0.07
9	Passing distance + sighting distance + number of observers + sighting distance * number of observers									88.40	1.54	0.07
10	Activity + passing distance + sighting distance									88.50	1.68	0.06
11	Age + passing distance + sighting distance + number of observers + reproductive category									88.60	1.79	0.06
12	Passing distance + sighting distance + number of observers + passing distance * number of observers									88.80	2.00	0.05
W_*i(V)*_:	Activity (0.13), age (0.32), passing distance (1.00), sighting distance (1.00), number of observers (0.57), reproductive category (0.29), passing distance * number of observers (0.05), sighting distance * number of observers (0.07)											

Resulting candidate models with ΔAIC_C_ < 2 after model dredging. We show AIC_C_ values, differences in AIC_C_ values (ΔAIC_C_) and AIC_C_ weights for models (W_*i(M)*_) and variables (W_*i(V)*_). The “+” symbol indicates inclusion of a variable in the models without an interaction with other variables, whereas the “*” symbol indicates interactions between the variables included.

**Table 4:** Best models explaining flight initiation distance (m) by brown bears in Sweden after being approached by humans.

Model		AIC_C_	ΔAIC_C_	W_*i(M)*_
1	Activity	180.90	0	0.50
2	Activity + age	181.80	0.96	0.31
3	Activity + number of observers	182.80	1.98	0.19
W_*i(V)*_:	Activity (1.00), age (0.31), number of observers (0.19)			

Resulting candidate models with ΔAIC_C_ < 2 after model dredging. We show AIC_C_ values, differences in AIC_C_ values (ΔAIC_C_) and AIC_C_ weights for models (W_*i(M)*_) and variables (W_*i(V)*_). The “+” symbol indicates inclusion of a variable in the models without an interaction with other variables, whereas the “*” symbol indicates interactions between the variables included.

**Fig 2:**
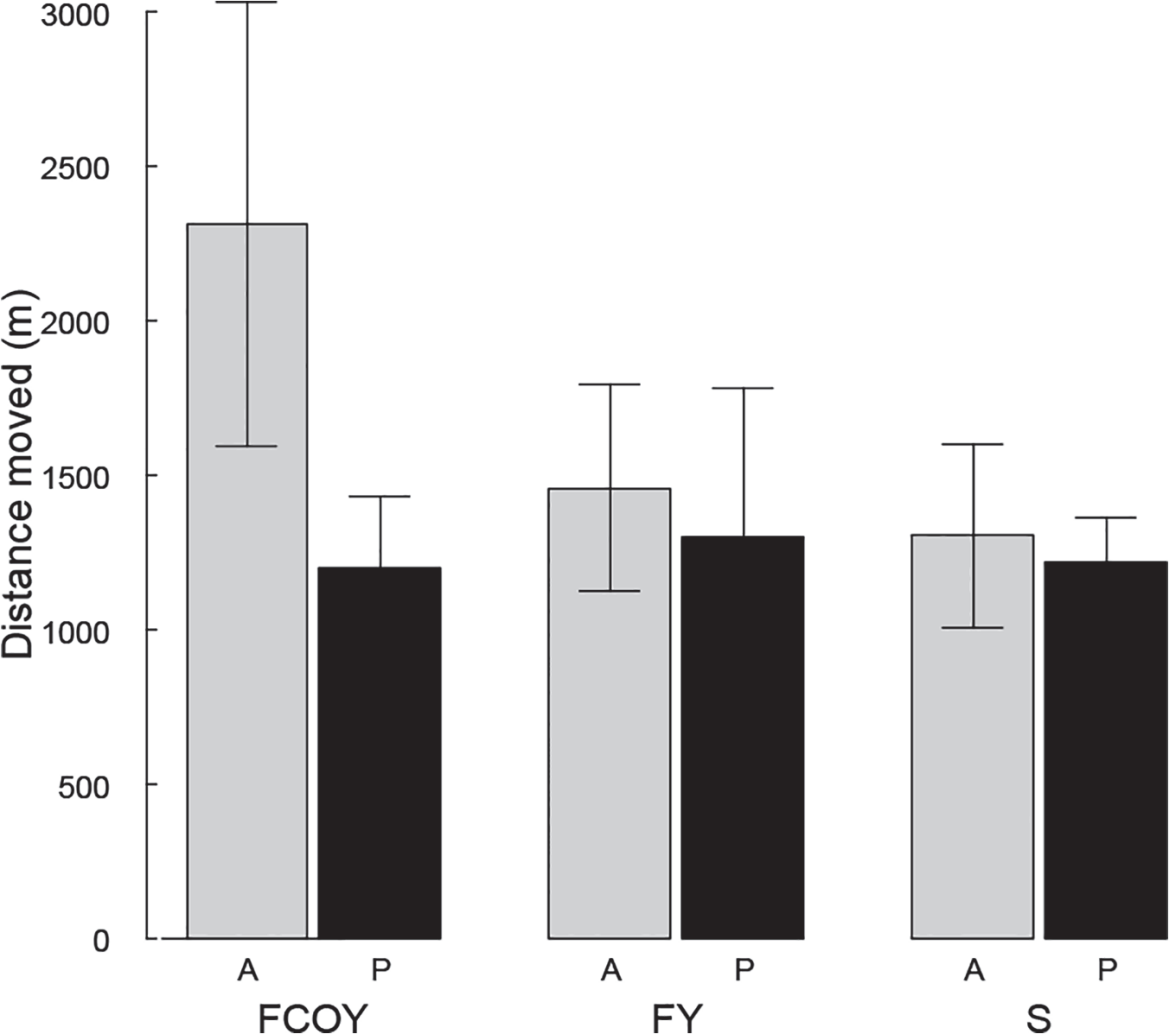
Distance moved (m) by Scandinavian brown bears after being approached by humans. Mean distance moved (m) after disturbance for active (A) and passive (P) Scandinavian brown bears in relation to family status (Fam Stat) with the categories: females with cubs of the year (FCOY), females with yearlings (FY), and single bears (S). The error bars show the standard error of the mean.

Bears that had been active at the start of the approach moved farther than those that had been passive and females with cubs moved farther than single bears, particularly active FCOY (Fig 2). Bears, irrespective of family status, tended to move shorter distances when the sighting distance at the initial site was longer (i.e. less cover). There was a weaker effect of number of observers and passing distance (Table 5). There were only small differences in time spent active after the approach, model selection suggested weak effects of activity, family status and to an even lesser extent, number of observers, where time spent active tended to be longer for FCOY than single bears and FY, and for active bears and in approaches with more observers (Table 6).

**Table 5:** Best models explaining distance moved (m) by brown bears in Sweden after being approached by humans.

Model		AIC_C_	ΔAIC_C_	W_*i(M)*_
1	Activity + family status + number of observers + sighting distance + activity *: family status	803.20	0.00	0.34
2	Activity + family status + number of observers + passing distance + sighting distance + activity * family status + passing distance: sighting distance	804.20	0.97	0.21
3	Activity + family status + number of observers + passing distance + sighting distance + activity * family status + passing distance: number of observers + passing distance * sighting distance	804.40	1.17	0.19
4	Activity + family status + number of observers + passing distance + sighting distance + activity * family status	805.00	1.77	0.14
5	Activity + family status + number of observers + passing distance + sighting distance + activity * family status + passing distance * number of observers	805.10	1.92	0.13
W_*i(V)*_:	Activity (1.00), family status (1.00), number of observers (1.00), passing distance (0.66), sighting distance (1.00), activity * family status (1.00), passing distance * number of observers (0.32), passing distance * sighting distance (0.40)						

Resulting candidate models with ΔAIC_C_ < 2 after model dredging. We show AIC_C_ values, differences in AIC_C_ values (ΔAIC_C_) and AIC_C_ weights for models (W_*i(M)*_) and variables (W_*i(V)*_). The “+” symbol indicates inclusion of a variable in the models without an interaction with other variables, whereas the “*” symbol indicates interactions between the variables included.

**Table 6:** Best models explaining time brown bears in Sweden spent active after being approached by humans.

Model					AIC_C_	ΔAIC_C_	W_*i(M)*_
1	-				264.10	0.00	0.23
2	Number of observers				265.20	1.15	0.13
3	Family status				265.20	1.16	0.13
4	Activity				265.50	1.43	0.11
5	Activity + family status				265.50	1.46	0.11
W_*i(V)*_: Activity (0.22), family status (0.24), number of observers (0.13)							

Resulting candidate models with ΔAIC_C_ < 2 after model dredging. We show AIC_C_ values, differences in AIC_C_ values (ΔAIC_C_) and AIC_C_ weights for models (W_*i(M)*_) and variables (W_*i(V)*_). The “+” symbol indicates inclusion of a variable in the models without an interaction with other variables, whereas the “*” symbol indicates interactions between the variables included.

## Discussion

We found no support for our prediction that females with cubs would leave earlier than single bears, as has been documented for family units in ungulates [39,40]. This may be because bear cubs have alternative defensive responses other than fleeing, such as climbing a tree. The vast majority of bears (95% of females with cubs and 88% of single bears) left following disturbance. Our results indicate that the risk of being discovered was a primary driver to leave the location. Activity at the time of the disturbance appeared to be the greatest determinant of the FID for both single bears and females with cubs, as previously documented [15]. Vegetation cover did not appear to affect the bear’s FID, which differs from what has been previously found for this study population [15], but the effect of cover on FID can differ between and within species [40] and may reflect individual variation in behavioural responses.

Females with dependent offspring select more open habitat at the landscape scale during the mating season than either lone females or adult males as a potential counter-strategy to sexually selected infanticide, but the difference in habitat selection was not as pronounced after the end of the mating season [41]. It is possible, however, that the difference in vegetation cover we documented between single bears and females with cubs at small habitat scales (Fig 2) is attributable to such social factors. Females with cubs may use more open habitat, because it is less preferred by other categories of bears that may pose a risk to the cubs, but also because more open habitat facilitates detection of potential threats. An adult bear without dependent young typically has little to fear from other bears; in fact, much of a single adult bear’s habitat selection at small and large scales appears to be directed at avoiding humans [7,14]. However, females with cubs must account for the threat of conspecifics, at least during the mating season, and evidence suggests that humans in this context may be the “lesser of two evils” [41,42]. An alternative, or additional, explanation to the use of open habitats by females with cubs is the greater berry abundance associated with more open habitats [43]. Berries are the primary source of food for Scandinavian brown bears at this time of year [44,45] and females may have to select feeding areas that will have enough food for both herself and her offspring, thus forgoing the shelter available in less open habitat.

The choice of more open habitat may also explain the trend of detecting females with cubs more often than single bears during the approaches. Despite this, none of the females with cubs displayed any aggressive behaviour towards the observers and all of the visually observed females with cubs fled the site after detection. In fact, none of the bears, whether females with cubs or singles, reacted aggressively to the observers, which reaffirms the results of previous studies [15,16]. This is an essential message for managers and forest users.

After disturbance, active and passive females with cubs and active single bears settled into denser habitats, which has also been documented in ungulates ([40] and references therein). However, passive single bears showed no difference in cover between initial and second sites. Moen et al. [15] proposed that the lack in difference in cover between initial and second sites for single passive bears was because they already had selected a protected site to rest. Passive females with cubs did not show this pattern, which further strengthens our conclusion that females with cubs took factors other than humans into account when selecting a daybed. The selection of a denser site following disturbance may indicate that females with cubs, once exposed to an approaching human, consider the human threat more in the selection of the habitat to which they retreated.

Active FCOY displayed the greatest reactions to disturbance, which is consistent with the predictions from the predation-risk hypothesis framework [21], indicating that bears do perceive humans as a threat. Female bears with dependent offspring may have to compromise in terms of habitat selection to account for both humans and conspecifics, but this does not lessen the impact approaching people have on them.

Less dense vegetation at the initial site reduced the strength of the responses in both females with cubs and single bears. Ordiz et al. [16] documented a similar effect of vegetation cover on the strength of the responses to the approach, particularly when the bears detected humans at short distances. More open habitat may provide bears with a better overview of the approaching threat, enabling them to assess the threat and the best course of action. This may mitigate the effect of the disturbance and diminish the strength of the bears’ responses. However, this also highlights the importance of dense cover for bears, which rely on dense locations for resting during daytime, when humans are outdoors [7].

We did observe an effect of the number of observers present during the approach for all the response variables under investigation. When there were more than 2 observers present, bears left in all approaches. There also appeared to be a greater disturbance effect with a higher number of observers. However, as we only had 2 approaches each of 4 and 6 observers, and 21 with 3 observers, we interpret these results cautiously.

### Are females with cubs more aggressive than solitary bears?

Aggressive defence of dependent offspring is a form of parental investment [46,47]. Increasing maternal aggression during the more sensitive offspring developmental stages has been suggested to improve protection of offspring against threats from conspecifics or predators [48]. Attacks on humans from females accompanied by dependent young has been documented in large carnivores [49,50] and ungulates [51,52]. Females with young are not typically the most frequent aggressors [1,49,50,52,53], but grizzly bears (also *U*. *arctos*) do not appear to follow the same pattern (see [54] and references therein).

Human injury rates and statistics confirm why female black bears *Ursus americanus* and brown bears with dependent young have a reputation for being more aggressive than their solitary conspecifics [55,56]. However, in our study, females with cubs were not more prone to defend themselves or their offspring more aggressively than other bears. Rather, our results suggest that underlying behavioural patterns moderated by social factors better explained the greater risk of injury to unarmed recreational forest users. This is because females with cubs often occupy areas closer to humans and human activity and their active periods overlap more with human active periods [14,42]. This, and the fact that females with cubs use more open habitat than single bears, makes them more likely to encounter humans that are in the forest for recreational purposes, such as berry or mushroom picking or hiking. Our results show that bears aware of approaching humans do everything they can to avoid an encounter. Moen et al. [15] showed that single bears tend to select daytime resting habitat that is so dense that people in the forest for recreational purposes are unlikely to enter it. However, the more open habitat used by females with cubs may make them the most likely bear category that recreational forest users would encounter.

Bear-human encounters occur infrequently [11] and in Scandinavia no fatalities have resulted from aggressive encounters with females with cubs for over 100 years (although one has been documented in Finland [57]). However, our study was not designed to assess any differences between single bears and females with cubs who had been surprised at close distances, as we approached bears talking with each other and passed the bears with the wind toward them. This consideration must be taken into account.

### Management implications

Incidents where humans have been injured or killed lead to decreased acceptance for large carnivores, including bears [12], and, besides increasing human safety, minimizing the risk of injury to people is also in the interest of management and conservation of large carnivore populations. The perception of risk has a major influence on the attitudes and behaviour of the public and wildlife managers [58,59], and fear is related both to a person’s uncertainty about their own responses to an encounter and to the perceived unpredictability of the animal [60,61]. Information that can provide recommendations about how people should behave to minimize risk, as well as provide them with knowledge that reduces animals’ perceived unpredictability has the potential to alter risk perceptions and reduce fear. This is likely to be a very effective tool in increasing tolerance for the species, or management action, in question. Our findings suggest that the best way for recreational forest users to avoid encountering a bear, regardless of its reproductive category, is to make noise while walking, to talk with each other or to oneself if alone and to pay attention to wind direction, especially if approaching patches of denser vegetation where bears usually rest during daytime.
